# An Anomalous Case on Salivary Calculus

**Published:** 1890-10

**Authors:** Edmund Owen


ARTICLE IX.

### AN ANOMALOUS CASE ON SALIVARY CALCULUS.

BY EDMUND OWEN, F. R. C. S.

A lady recently came under my care for a troublesome but painless swelling of the left cheek. The medical man who sent her to me suggested that it was a case of distention of a greatly dilated parotid duct, possibly caused by a salivary calculus. Unfortunately this gentleman did not accompany her on her visit to me, and on the most careful examination I could detect no calculus, but found the cheek of that side somewhat prominent and unsightly. The lady said that she had been bothered by a swelling in that cheek ever since she was four years old, and that sometimes after a meal it was much more conspicuous than it was at another time. She was determined to have something done for the swelling, and I was content to hold my diagnosis as to the exact nature of the soft tumour in the suspense until I could make a thorough exploration, which I did a few days later, Dr. Prickett kindly helping. There was then a rounded doughy swelling of the one cheek, but no calculus was discoverable. Anæsthesia having been produced, and the jaws being separated by Mason's gag, an incision was made through the mucous lining of the mouth and the buccinator, a lobulated piece of yellow fat at once protruding. Gentle traction being made on this, a lipoma of considerable size readily left its bed between the buccinator and masseter muscles. The cheek was then flat like the other. It seemed as if no further treatment would be required, and nothing more was attempted. Within a few days, however, it became evident that the patient did not share our favorable view of the case. She said that on several occasions the cheek had swollen as badly as ever. Never happening to see the cheek, however, when swollen as she described, I thought it not improbable that her imagination supplied her with such evidence as I failed to discover. She was therefore advised to return home and to apply again should she meet with further inconvenience. In a short while she duly presented herself, and directed attention to a small, hard substance, which shifted its position over the masseter; it was evidently the salivary calculus of which her medical attendant had spoken. An attempt was promptly made to extract it through the mouth by reopening the old wound, but on introducing a pair of forceps the concretion slipped away and so effectually concealed itself that further search for it on that occasion had relucantly to be abandoned. On a subsequent occasion on which the calculus was discovered I leisurely examined it from the outside of the cheek, and found that the limit of its journey forwards was just beyond the hinder border of the masseter, and that with the slightest touch it slipped back into a dilatation of Stenson's duct, which formed a wide chamber behind the angle and ramus of the jaw. From this pouch the calculus could be swept out by firm pressure. Sometimes it was no easy matter to bring it out again, as it hid itself on the inner aspect of the mandibular angle in the capacious chamber. Having been twice disappointed in the treatment of the case when operating through the buccinator, I determined to cut straight down on to the calculus through the cheek, having chased it forwards and secured it by the finger pressed over the hinder part of the masseter. In this way its extraction proved a simple matter. The skin wound, which was closed with horsehair sutures, healed by first intention, giving no trouble whatever as regards leakage of saliva, and leaving a scar which, from the patient's point of view as well as the surgeon's, is now hardly noticeable. The calculus was a phosphatic concretion of the size and shape of a small date stone.

*Remarks.*—No one who has carefully dissected the face can fail to have noticed the pad of yellow fat which is lodged between the masseter and buccinator, the little pellets of which obeyed the slightest touch of his forceps. But in the case under consideration the mass of fat far exceeded the normal amount, and when drawn out through the mouth constituted a lipoma of a very respectable size. Moreover, the swelling which had previously disfigured that cheek had so entirely disappeared that I feel justified in saying that the operation would prove entirely successful. Probably the irritation caused by the salivary calculus had brought about an over-nutrition of the fatty pad and so determined its hypertrophy, the other cheek remaining of normal size and appearance. Certainly the removal of the calculus alone would not have restored the symmetry, and had I extracted the concretion on the first occasion I might have hesitated to proceed to the ablation of the buccal lipoma, even if I had made a correct diagnosis of the nature of that swelling. Thus, as possibly not infrequently happens, failure on the part of the surgeon either in the way of diagnosis or treatment, worked for the good of the patient. As regards the removal of the calculus from the outside of the cheek, it is not an operation which one would generally recommend, lest a troublesome salivary fistula should result, but, seeing the perfect way in which the wound healed, the danger of such a contingency is probably over-rated.—*Lancet.*

